# Informal Coping Strategies Among People Who Use Opioids During COVID-19: Thematic Analysis of Reddit Forums

**DOI:** 10.2196/32871

**Published:** 2022-03-03

**Authors:** Josh Arshonsky, Noa Krawczyk, Amanda M Bunting, David Frank, Samuel R Friedman, Marie A Bragg

**Affiliations:** 1 Department of Population Health New York University Grossman School of Medicine New York, NY United States; 2 Behavioral Science Training Program in Drug Abuse Research New York University New York, NY United States

**Keywords:** opioid use, Reddit, coping strategies, COVID-19, opioid, drug, coping, social media, strategy, content analysis, abstain, addiction, data mining, support

## Abstract

**Background:**

The COVID-19 pandemic has transformed how people seeking to reduce opioid use access treatment services and navigate efforts to abstain from using opioids. Social distancing policies have drastically reduced access to many forms of social support, but they may have also upended some perceived barriers to reducing or abstaining from opioid use.

**Objective:**

This qualitative study aims to identify informal coping strategies for reducing and abstaining from opioid use among Reddit users who have posted in opioid-related subreddits at the beginning of the COVID-19 pandemic.

**Methods:**

We extracted data from 2 major opioid-related subreddits. Thematic data analysis was used to evaluate subreddit posts dated from March 5 to May 13, 2020, that referenced COVID-19 and opioid use, resulting in a final sample of 300 posts that were coded and analyzed.

**Results:**

Of the 300 subreddit posts, 100 (33.3%) discussed at least 1 type of informal coping strategy. Those strategies included psychological and behavioral coping skills, adoption of healthy habits, and use of substances to manage withdrawal symptoms. In addition, 12 (4%) subreddit posts explicitly mentioned using social distancing as an opportunity for cessation of or reduction in opioid use.

**Conclusions:**

Reddit discussion forums have provided a community for people to share strategies for reducing opioid use and support others during the COVID-19 pandemic. Future research needs to assess the impact of COVID-19 on opioid use behaviors, especially during periods of limited treatment access and isolation, as these can inform future efforts in curbing the opioid epidemic and other substance-related harms.

## Introduction

In 2020, with the arrival and devastation of the COVID-19 pandemic, many of the efforts in fighting the preexisting opioid epidemic in the United States were interrupted and the number of opioid-related deaths have since accelerated. Reports from national, state, and local agencies have shown an alarming increase in opioid-and other drug-related mortality—especially from illicitly manufactured fentanyl [1]. In 2021, overdose deaths soared to a record estimate of 93,331, including 69,710 (74.69%) involving opioids [2]. The overall estimate of overdose deaths in 2020 far eclipsed the 72,000 deaths recorded the previous year, amounting to a 29.4% increase.

COVID-19 has affected nearly every facet of society, and it has seriously burdened people who use opioids (PWUO) and people who use drugs (PWUD) in general. Health experts are concerned how the public health measures responding to the pandemic—particularly quarantine and social distancing rules—may have exacerbated active drug use and drastically changed remission statuses on the population level [3,4]. PWUD are at an increased risk of COVID-19 transmission and infection and are more likely to be hospitalized for severe symptoms [5,6]. Given the severity of this ongoing public health crisis and the likelihood that further pandemics will take place in coming decades [7-13], it is imperative to understand how PWUO have dealt with the physical and psychological changes brought on by the COVID-19 pandemic, particularly in response to stay-at-home orders that limit in-person interactions.

Only a small percentage of PWUO seek formal treatment options, with less than 25% of PWUO estimated to use treatments with evidence-based medications for opioid use disorder (MOUD) [14]. Others may turn to strategies such as social media for health-related information and community support [15]. Online social media platforms enable PWUD to freely share their experiences, give advice, and comment on others’ experiences. Studies have shown that for PWUD, online peer support groups provide an open forum for discussion, while minimizing perceived barriers and stigma [16]. In the context of the COVID-19 pandemic, it may serve as a vital source of personal narratives of opioid use and a means to cope with opioid use without formal assistance (ie, informal coping) or seek formal assistance through treatment (ie, formal coping).

Many barriers exist that may prevent PWUO from seeking formal treatment. Social networks, for example, are powerful influences among PWUD that can encourage continued use or serve as a catalyst for return to use [17,18]. Stigma from both health care workers [19] and family members and friends [20,21] may discourage PWUD from disclosing their substance use disorder and receiving treatment. Access to evidence-based MOUD treatments, such as buprenorphine and methadone, are limited by regulations that require in-person examinations, and physician waivers for buprenorphine prescriptions [22] and take-home allowance limits for methadone result in daily in-person visits to clinics [23]. Finally, PWUO may want to discontinue use, but fear the resulting withdrawal symptoms, which can be painful and distressing [24]. The potential length of withdrawal may inhibit PWUO from cessation if they cannot risk losing work or have other obligations that they must fulfill [24].

Although the COVID-19 pandemic has exposed PWUO to new risks (eg, possible triggers due to loneliness, boredom, and isolation, as well as forcing people into withdrawal due to lack of access), behaviors that prevent the spread of COVID-19 may help mitigate barriers to cessation or entry to treatment. The change in environment spurred by social distancing measures may make it easier for some PWUO to avoid influences in their social circle that might encourage them to continue using drugs. Avoiding these influences might ease unwanted pressures while they make decisions regarding cessation or treatment [17,18]. Still, a large portion of PWUO do not seek formal treatment, making it critical for the clinical and public health field to understand how people outside of the clinical setting cope with opioid use, especially now within the context of the COVID-19 pandemic.

Using data collected from Reddit, this exploratory and descriptive study aims to characterize the types of informal coping strategies people used to reduce or abstain from opioid use during the first wave of the COVID-19 pandemic in 2020.

## Methods

### Study Design

This study is part of a larger project that sought to understand the experiences of PWUO during the first wave of the COVID-19 pandemic [25,26]. We extracted publicly available content from Reddit, an anonymous and community-based online platform dedicated to public discussions. Anonymous registered members—also called *Redditors*—submit content to the site, including text, images, or links, which are then filtered by popularity as they are voted up or down by other members. Reddit posts are organized by subject and grouped into user-generated forums called *subreddits*. Posts with more up-votes appear toward the top of their subreddit.

Reddit has over 430 million monthly active users, and in 2020, it was ranked the seventh-most popular social media app in the United States [27]. Because of its anonymity, Reddit provides a virtual space to exchange information about stigmatized topics, such as drug use and mental health [28,29]. This, in turn, provides researchers with a unique opportunity to gain, in real time, insights into behaviors and perceptions that can inform health and drug policy. The anonymity of Reddit, however, hinders our ability to retrieve demographic information of Redditors’ whose posts we have included in our analysis. However, in many of our sampled subreddit threads, Redditors disclosed their personal history of opioid use and whether, at the moment of posting, they were abstaining from use. So, despite the lack of demographic information on Redditors, the analyses presented in this exploratory study can still inform efforts to advance health and drug policy—albeit to a certain extent—during the COVID-19 pandemic.

As researchers of drug use and social media, we were interested in characterizing the informal ways in which PWUO navigated the disruptions caused by the first wave of COVID-19 in 2020. Though none of the authors subscribe to the subreddits analyzed in this study, 3 of us have used Reddit in the past and are familiar with the platform and communities it coalesces. At each stage of the study, we discussed our methods, findings, and interpretations with individuals who have lived experiences with the subject matter. The New York University School of Medicine Institutional Review Board exempted our study from review. To protect the anonymity of Redditors, quotes were altered slightly to reduce the risk of immediate identification [30,31].

### Collecting Reddit Data

In this study, we analyzed posts from the 3 most popular subreddits about opioids: *r/Opiates* and *r/OpiatesRecovery*. We determined their popularity by the number of Redditors subscribed to each subreddit. At the start of data collection, in March 2020, *r/OpiatesRecovery* had more than 20,000 subscribers; *r/Opiates* had over 90,000.

To pull content from each subreddit, we used Python 3.7.4 run on Jupyter Notebook 6.0 using packages *PRAW, pandas*, and *datetime*. On May 13, 2020, we extracted the 1000 most recent posts—including their subsequent comments—from each subreddit. This amounted to a total of 2000 unique subreddit posts with comments, all of which Redditors posted from March 5 to May 13, 2020. After pulling the posts, we stored them in an online, password-protected database.

### Reddit and Qualitative Coding Training

We first trained 9 research assistants on how to conduct qualitative coding using Atlas.ti software (ATLAS.ti Scientific Software Development GmbH). Those 9 coders then underwent 4 one-hour training sessions—developed by our team—on medical terms and slang related to opioid use and Reddit culture.

### Thematic Analysis

The 9 trained coders then reviewed all 2000 subreddit posts to determine whether they were relevant to COVID-19. To determine relevance, we used the following list of key terms: *coronavirus*, *COVID-19*, *quarantine*, *social distancing*, *shutdown*, *pandemic*, and other related terms referring to the pandemic. To initially identify COVID-related content, coders reviewed only the original subreddit posts and not their subsequent comments. We wanted to avoid situations in which coders reviewed threads of 50 or more comments, only to discover that 1 comment related to COVID-19. Coders identified 300 (15%) original subreddit posts related to COVID-19; we discarded the remaining 1700 (85%) posts because none of them explicitly included our keywords or hinted at any aspect of the pandemic and its effects.

Using a hybrid inductive-deductive approach through Atlas.ti, the 9 research assistants then coded the 300 COVID-19-relevant subreddit posts and comments using a codebook we created prior to data collection [25,26]. The codebook spanned study themes of interest related to the intersection of opioid use, treatment, and COVID-19. Research assistants coded an initial 30 (10%) of the 300 posts. Afterward, we met with them to assess coding discrepancies and add any new or common topics identified from the subreddit posts to our codebook. We used the methodological approach described by McDonald et al [32]. They specify that for research focused on discovering new concepts, codebook development meetings do not aim to achieve a certain threshold of agreement or interrater reliability. Instead, the goal of the codebook development discussions is to generate concepts and themes that enable discovery of novel content for an exploratory study. During the training process, we asked coders to identify any terms that were unfamiliar to them. Then, during group meetings, we defined these terms—which were often slang terms. The final codebook had 44 codes (see Table S1 in Multimedia Appendix 1), and research assistants coded the remaining sample of COVID-19-related posts.

Using solely an inductive approach, the first author analyzed the 300 COVID-19-related posts previously coded by research assistants to identify and characterize Redditors’ informal coping strategies during the early weeks of the COVID-19 pandemic in 2020. Based on previous addiction and recovery literature [33], *informal coping strategies* were defined as those that did not involve professionally assisted support services, such as outpatient/inpatient treatment, antirelapse/anticraving medications (eg, naltrexone or buprenorphine/naloxone), mutual help groups (eg, Alcoholics Anonymous [AA], Narcotics Anonymous [NA], Self-Management and Recovery Training [SMART]), and other community-based support where trained staff typically provide services [33]. Of the 44 codes, 16 (36%) were used to filter out posts relevant to informal coping strategies (see Table S2 in Multimedia Appendix 1). The first author discarded subreddit posts that were specific to other research questions (eg, formal treatment services, such as medication-assisted treatment) or if the content did not include or was entirely unrelated to informal coping strategies to maintain remission or begin/continue cessation from opioid use.

After meeting with coauthors and agreeing on the major themes characterizing the informal coping strategies described in posts, the first author scanned nonexamined Reddit content to identify those same strategies and skills. Using the search function feature, he searched through previously unexamined posts for the exact terms or phrases listed in Table 1. After examining the content’s relevance to our research aims, the first author either added it to our preexisting sample of content or discarded it. This process resulted in an increase of 12 subreddit posts, making the total sample amount to 100 posts.

**Table 1:** Frequency of informal coping strategies described by Redditors during the first wave of the COVID-19 pandemic in 2020 (N=100).

Themes		Posts, n (%)
**Psychological/behavioral coping skills (eg, psychological and emotional challenges, triggers)**		
	Mindfulness (eg, reframing personal problems, identifying discontentment)	20 (20)
	Meditation	5 (5)
	Prayer	2 (2)
	Gaming (eg, video games, board games, internet-based games)	12 (12)
	Reading books	4 (4)
	Listening or making music	4 (4)
	Household chores	1 (1)
	Watching movies, television, or videos on social media	6 (6)
	Calling friends and family	6 (6)
	Posting in subreddit or other online forums (eg, encouraging others to post online, disclosing struggle)	5 (5)
	Taking up hobbies or learning new skills	9 (9)
	Total	74 (74)
**Adopting healthy habits**		
	Exercise	26 (26)
	Drinking water	2 (2)
	Eating healthy	6 (6)
	Sleep hygiene	4 (4)
	Total	38 (38)
**Using other substances to manage withdrawal symptoms**		
	Marijuana	9 (9)
	Transcutaneous electrical nerve stimulation (TENS) device (ie, device used to stimulate stiff muscles)	1 (1)
	Liposomal vitamin C	1 (1)
	Kratom	6 (6)
	NyQuil	1 (1)
	Total	18 (18)

## Results

### Informal Coping Strategies

Of the 300 subreddit posts included in our sample, 100 (33.3) posts mentioned at least 1 type of informal coping strategy. Table 1 shows the frequency of Redditors’ posts that described at least 1 type of informal coping strategy during the pandemic. Examining these messages revealed a myriad of strategies people used for remission and recovery from opioids. In total, there were 4 types of informal coping strategies that Redditors used themselves or suggested to others. The most frequent were psychological and behavioral skills (74/100, 74%), followed by adopting healthy habits (38/100, 38%) and using other substances to manage withdrawal symptoms (18/100, 18%).

#### Psychological and Behavioral Coping Skills (n=74, 74%)

The first wave of the COVID-19 pandemic upended the ways people worked and lived. Many employers abruptly required employees to work remotely, and others were forced to lay off large numbers of staff. Quite suddenly and unexpectedly, many Redditors found themselves mostly restricted to their homes—forcing some to reevaluate their work-life balance and forcing others to navigate social distancing and stay-at-home orders without active incomes. Compounding these difficulties was effectively managing reduction in, or cessation of, opioid use while stuck at home.

To confront these particular challenges, Redditors described a variety of psychological and behavioral coping strategies. Although never explicitly stated, practicing mindfulness was a common psychological skill described in our sample of Reddit posts. According to previous work, mindfulness is generally described as a practice to bring awareness of and attention to the present moment without judgement [34]. To be mindful means to observe what is happening—both inside and outside yourself—in the here and now, without evaluating your observations [35]. Redditors described their mindfulness practices in some of the following ways:

I try to look for the good things when I’m sober, the chirps of birds, the sound of trees in the wind, and the warmth from the sun on my skin always comes back. It’s great to finally be able to listen to the world when I’m sober.

Sobriety can be boring but learning to cope with your baseline emotions feels so much better than dealing with the intense highs and lows of opiates. It’s not natural and not worth it.

I make videos every day and write down just how fucking awful I feel to go back and really remind myself how bad it is and why I don’t ever want to do it again once I’m done.

The subreddits themselves served as a psychological coping strategy in that they provided Redditors with an easily accessible space for communal support. Redditors shared their experiences and strategies—both successful and unsuccessful—with others to encourage their cessation of or reduction in opioid use, supporting and empowering them to push forward and continue making progress:

I made a plan that was damn near [foolproof] where no matter what happened or how bad it got I had a contingency in place to keep me from getting in my car and going and copping. I used that plan more than I thought I would and I’m glad I had it. It included things like calling my brother or sober friends or when I knew I was going to have a tough day I’d just go spend it with my dad or at the animal shelter . . .

In some instances, Redditors posed thoughtful questions to those who disclosed their personal struggles:

You’re also doing something important in reaching out and venting on here, those connections are so important, it certainly doesn’t sound like dumb bullshit. Sounds like you’ve got a lot of inner strength from previous experiences, and you can acknowledge what you’ve learned from them. Maybe part of coping with this is asking what you can learn from this new thing (in a way you are already asking that!), but I know it’s really hard.

Redditors exchanged many behavioral strategies to combat boredom and distract themselves to reduce or abstain from opioid use amid sudden COVID-19 circumstances. The most frequently discussed behavioral strategy was gaming via PlayStation and other consoles, as well as via board games and different websites.

Other Redditors distracted themselves at home by picking back up old hobbies and activities or learning new ones:

I’m not working right now so I’m at home by myself for most of the day, until my husband gets home from work. I’m taking advantage of it though. I’m working on a master list of activities around the house I can do that will help me distract from any paranoid thoughts. Right now my list is made up of music-related things (singing, playing ukulele) and learning-related things (continuing an online class I started a month or so ago) and craft-related things (working on a diamond painting, picking up knitting again).

In some posts, Redditors mentioned watching movies and amusing videos on social media to fight boredom. For 1 Redditor, an effective strategy to redirect their negative thinking of their personal hardship was to watch documentaries that reframe such hardship through context and comparison:

. . . I’m super weird in that when I’m kicking sometimes I like to watch documentaries about really fucked up shit to remind me that like my situation isn’t that bad I guess? Lol kinda crazy but it does work for me in terms of just getting my mind out of that horribly dark place . . .

For 1 self-disclosed “sober” Redditor, using marijuana became another important and much-needed tool to combat boredom and maintain remission from opioid use after withdrawal symptoms dissipate:

Pot is a lifesaver for me in sobriety and I’m not even a pot guy really, it helps me with relative boredom that comes with not chasing pills and just sort of allows me to focus on other things. Doesn’t do a damn thing for me in active [withdrawals] but post [withdrawals] it's a solid maintenance tool.

#### Adopting Healthy Habits (n=38, 38%)

On both subreddits, during the early days and weeks of the COVID-19 pandemic in 2020, Redditors also emphasized the crucial role of developing healthier habits—such as exercising, staying hydrated, and eating healthy—to help improve mood and overall well-being. Some Redditors shared their healthy practices for getting more energy throughout the day or for clearing brain fog:

Stay at home but go for a walk or run, drinks and drinks and drinks to stay hydrated, consume funny media, take a nice shower, clean your tub . . . I need to. But get enough sleep exercise and try to eat okay and you'll start thinking clearer and things will get better.

I’ve been focusing on getting more energy every day. It’s been pouring rain where I am for the last few days which sucks [because] I was getting back into jogging and it feels so good!! And I’ve been doing yoga pretty regularly, too!!

In some instances, Redditors not only described their routines but also disclosed their gradual health effects in real time. For example, 1 Redditor tracked their progress of adopting healthy habits and the impact it had on their sleep:

Wanted to die in week [1], started eating clean in week 2, started exercising/lifting/getting hobbies back in week 3, and sleep started returning to (almost) baseline in week 4. Things are starting to look up again and I can’t wait [till] quarantine is over.

#### Using Other Substances and Devices to Manage Withdrawal Symptoms (n=18, 18%)

Redditors shared information about managing withdrawal symptoms. In some cases, they used definitions of “sobriety” that allowed for certain kinds of substance use (eg, marijuana) but not others:

I stay clean by hitting my THC vape, and by playing video games or making music. I’m not doing hard drugs because my fiancé is stuck at home with me. She would be very upset if I did.

For clinicians and public health experts, this serves as a valuable reminder that definitions of “sobriety” and acceptable substances may differ across patients.

One Redditor discussed using transcutaneous electrical nerve stimulation (TENS) to help with restless legs and muscle pain during withdrawal. These posts referred to COVID-19 allowing the use of alternative substances and devices to help manage opioid use.

So I decided to make my first post here, [m]aybe it will help someone! There is this device for muscle problems: Transcutaneous electrical nerve stimulation (TENS) is a method of pain relief involving the use of a mild electrical current. Some people use it to train their muscles without actually training but I don’t know if it really works. I personally got it prescribed because of a muscle problem on my neck! It basically gives you a massage and for that purpose its ideal! But I was also able to use a TENS device successfully against my restless legs while withdrawing! If you want to know how it looks just google: Tens/EMS device/unit.

### Using Quarantine as an Opportunity for Opioid Cessation or Reduction (n=12, 4%)

Of the 300 subreddit posts included in our sample, 12 (4%) posts mentioned using quarantine as an opportunity for cessation of or reduction in opioid use. Redditors acknowledged that despite the stress caused by COVID-19, the pandemic represented an ideal opportunity to work toward cessation or reduction. The 12 posts referred to quarantine, remote work, and reduction in responsibility as valuable circumstances for their recovery efforts.

Anyway, as insensitive as it sounds, corona plopped in my lap at a perfect time. It gave me the perfect opportunity to detox and start recovering without any real responsibility.

You still have options, and the [COVID] shutdown is a great excuse to be able to take a few weeks away from the world and ride out the withdrawals.

Thankfully, this pandemic has given me an opportunity to begin recovery. But I know I’m one of the lucky ones, as boredom doesn’t get to me.

## Discussion

### Principal Findings

This exploratory study sought to characterize some of the informal coping strategies used by people who were abstaining from use—or simply managing their opioid use as best as they could—while adjusting to the COVID-19 pandemic and remaining at home during the first wave in 2020. To do this, we thematically analyzed a sample of subreddit threads posted on Reddit sometime between March and May 2020. In 12 (4%) of 300 posts, Redditors remarked that the pandemic provided them with an unparalleled opportunity to reduce opioid use. Our analyses revealed 3 overarching themes of informal coping strategies: psychological and behavioral coping skills (74/100, 74%), adopting healthy habits (38/100, 38%), and using substances to manage withdrawal symptoms (18/100, 18%). To help distract themselves from “negative” thinking and the desire to use opioids, Redditors used a variety of strategies. These included activities such as playing video games, taking up old hobbies again, or learning new ones. Redditors also described their adoption of healthy eating habits and exercise and mindfulness practice. In some instances, Redditors disclosed using substances such as marijuana to sway boredom or manage withdrawal symptoms. These exploratory findings may help further our understanding of how the first wave of COVID-19 uniquely affected PWUO, especially people seeking to reduce their opioid use. Ultimately, contributing to this knowledge base will help inform future drug policy and harm reduction efforts responding to future disruptive public health emergencies.

### Limitations

Our study, however, is not without limitations. First, the majority of Redditors are younger men living in the United States [36], so the strategies of coping reflected in the subreddit threads may not represent strategies used by the larger community of PWUO. Second, because of the anonymity of Reddit, we had no demographic information or history of drug use for people who posted on both subreddits, so we could not examine the extent to which suggested coping strategies were effective in recovery. Another limitation of our study is that we were not able to verify demographic characteristics and the exact location of the Redditors. It is possible that some of the posts were written by PWUD or their friends or family members. To address this limitation in future studies, researchers could survey patients who are in treatment for OUD and ask them if they have ever used Reddit to discuss opioid use. Third, we did not evaluate the intercoder reliability of our coding frames. We fully believe in the practice and acknowledge its many benefits for qualitative analysis. These benefits include improving the systematicity, communicability, and transparency of the coding process; promoting reflexivity and dialogue among researchers; and helping convince diverse audiences of the trustworthiness of the analysis [37]. With this research, however, we followed the guidelines set by McDonald et al [32]. We did not aim for a certain threshold of agreement. Rather, we sought to generate concepts and themes so that novel content could be discovered and tested in future exploratory studies. Fourth, future studies should return to subreddit threads to examine data during a longer time frame. Our aim with this study, however, was to understand Redditors’ conversations during the first moments of the pandemic. Analyzing their dialogue during the acute time frame enabled us to examine their coping skills during a crisis. Finally, it is unclear whether these Redditors were a highly motivated group that would have succeeded without support from a social media site; it is possible that other social media sites facilitate forums that exacerbate risk factors for opioid use.

### Future Implications

These data have several implications for treatment providers, specifically. First, in times of crisis when social interaction is limited, providers can encourage patients to use online forums as a coping strategy and source of communal support. Second, health care workers can listen nonjudgmentally to patients who may describe Reddit as a valuable recovery tool, and be mindful that it represents a safe space for patients who value to anonymity of the forum. Social media and other forums are popular resources for health information, even though some forums may spread misinformation [38]. Studies have shown that people may seek forums and self-help groups to provide emotional support [39-42], particularly given online forums may allow anonymous disclosure without feelings of shame or guilt [43]. Finding emotional support without being stigmatized is especially relevant for PWUD [44] and is an urgent matter, given opioid overdose–related use of health care has increased throughout the pandemic. Future research should examine subgroups of PWUO who may be at heightened risk for reduced access to care during the pandemic [44].

### Conclusion

Our current analysis of subreddits supports our previous research suggesting that Reddit facilitates social support that may aid people in their efforts to reduce or abstain from opioid use and improve their physical and mental health [25]. In other research using the same original sample of Reddit content, we found that Redditors sought support and advice from each other on ways to access formal treatment for opioid use during the pandemic [26], whereas a different set of qualitative analyses of these data revealed that some Redditors reported increases or changes in active drug use (D. Frank, PhD, unpublished data, July 2021). Taken together, these findings demonstrate that Reddit is a valuable data source for rapid public health surveillance, especially for emerging public health crises, such as COVID-19.

## Appendix

Multimedia Appendix 1 Final codebook and list of codes used to filter out content for thematic analysis.

## Abbreviations

MOUD: medication for opioid use disorder
OUD: opioid use disorder
PWUD: people who use drugs
PWUO: people who use opioids
