# Supplementary material for: Informal Coping Strategies Among People Who Use Opioids During COVID-19: Thematic Analysis of Reddit Forums
Source: JMIR Form Res. 2022 Mar 3;6(3):e32871. doi: 10.2196/32871 (PMC8896559; doi:10.2196/32871)
Supplement: Multimedia Appendix 1 [file formative_v6i3e32871_app1.docx]

**MULTIMEDIA APPENDIX 1**

| **Table S1.** Final codebook originally proposed in our larger project | |
| --- | --- |
| **Code** | **Code Description** |
| Accessing telehealth services | Discusses switch to telehealth platforms to receive opioid treatment meds or other services related to COVID-19 closures |
| Changes in access to MOUD | Discusses issues related to accessing opioid treatment medications (methadone, buprenorphine, suboxone, or naltrexone) during COVID-19 |
| Stocking supply of medication | Discusses stocking or assuring full supply of medication treatment as to not run out during COVID-19 |
| Asking for advice/support | Asking other Redditors for advice or support related to issues arising from COVID-19 |
| Increased interest in treatment | Discusses new or increased interest in treatment as a result of COVID-19 circumstances |
| Challenges of remote work | Generated by coders |
| Changes in access to social networks | Discusses changes in communication and contact with friends, family, and others who comprise a person's support system |
| Changes in risk behaviors | Discusses changes to drug-related risk behaviors due to COVID-19 disruptions |
| Changes in dealer or regular drug supply | Discusses changes in access to drug supply tied to COVID-19 disruptions |
| Changes in drug use quantity/tolerance | Discusses changes in amount of drugs used because of COVID-19 circumstances |
| Changes in access to naloxone | Discusses problems accessing naloxone during COVID-19 |
| Changes in access to harm reduction supplies | Discusses problems accessing harm reduction supplies other than naloxone during COVID-19 (e.g., clean needles, fentanyl test strips) |
| Coping strategies for sobriety | Discusses self-strategies to reduce or abstain from opioid use during COVID-19 |
| COVID-19 study | Discusses opportunity to participate in COVID-19-related research study |
| COVID-related social distancing | Discusses experiences with COVID-19-related social distancing/quarantine/isolation |
| COVID-related closure changes | Discusses issues related to closure changes in service availability due to COVID-19 |
| COVID-related job/income loss | Discusses issues related to job loss due to COVID-19-related disruptions |
| COVID-related infection/illness | Discusses issues related to COVID-19 infection/illness and how it has affected them and/or their loved ones |
| Drug testing/probation | Generated by coders |
| Discussing new ideas | Discusses new thoughts and asks new questions that arise during the first wave of COVID-19 |
| Engaging in social support via online platforms | Discusses engaging with social support online as a way to confront the difficulties brought about by COVID-19 |
| Essential worker desiring hazard pay | Generated by coders |
| General difficulty of COVID-19 pandemic | Generated by coders |
| Gem quotes | Quote that sticks out as particularly compelling or interesting; it captures main themes of study |
| Helping others | Discusses actions or recommending sources to help others confront their personal challenges during COVID-19 |
| Increased fear or anxiety | Discusses feelings of fear and anxiety in response to COVID-19 circumstances |
| Increased interest in treatment | Discusses desire to engage in treatment for drug use because of COVID-19 circumstances |
| Law enforcement/criminal justice encounters | Discusses experiences related to criminal justice or encounters with law enforcement due to COVID-19 |
| Lack of distraction/boredom | Discusses lack of distraction and feelings of boredom tied to social distancing |
| Not related to COVID-19 | Does not discuss anything related to COVID-19 |
| Offering advice/support/information | Offers advice on navigating the pandemic or provides helpful information to those who seek it |
| Relapse/return to use | Discusses experiences with relapse in response to COVID-19 circumstances |
| Overdose | Discusses experience of overdose tied to COVID-19 circumstances |
| Reducing overdose risk | Discusses experiences related to reducing overdose risk, such as not using opioid alone or stocking naloxone despite COVID-19 changes |
| Side effects of drug use | Generated by coders |
| Stocking supply of drugs to avoid running out | Discusses stocking or assuring fully supply of illicit drugs so as to not run out and risk withdrawal during COVID-19 |
| Stream of consciousness/venting | Shares personal experiences and/or feelings about COVID-19-related struggles |
| Triggers for use/cravings | Discusses reminders of using drugs, cravings, etc. |
| Using quarantine to get sober | Generated by coders |
| Wearing protective gear | Discusses use of gear like masks and gloves to protect themselves from exposure to COVID-19 |
| Washing hands/hygiene | Discusses washing hands or other hygienic activities for protection from COVID-19 |
| Disinfecting drugs/other materials | Discusses actions related to disinfecting drugs or other exposed items |
| Using clean needles | Discusses use of clean needles despite COVID-19 disruptions |
| Withdrawal | Discusses experiences with withdrawal connected to COVID-19 circumstances |
|  | |

| **Table S2.** Excerpt from codebook with codes used to filter out relevant posts for thematic analysis | |
| --- | --- |
| **Code** | **Code Description** |
| Challenges of remote work | Generated by coders |
| Changes in access to social networks | Discusses changes in communication and contact with friends, family, and others who comprise a person's support system |
| Coping strategies for sobriety | Discusses self-strategies to reduce or abstain from opioid use during COVID-19 |
| COVID-related social distancing | Discusses experiences with COVID-19-related social distancing/quaratine/isolation |
| Discussing new ideas | Discusses new thoughts and asks new questions that arise during the first wave of COVID-19 |
| Engaging in social support via online platforms | Discusses engaging with social support online as a way to confront the difficulties brought about by COVID-19 |
| General difficulty of COVID-19 pandemic | Generated by coders |
| Helping others | Discusses actions or recommending sources to help others confront their personal challenges during COVID-19 |
| Increased fear or anxiety | Discusses feelings of fear and anxiety in response to COVID-19 circumstances |
| Lack of distraction/boredom | Discusses lack of distraction and feelings of boredom tied to social distancing |
| Offering advice/support/information | Offers advice on navigating the pandemic or provides helpful information to those who seek it |
| Relapse/return to use | Discusses experiences with relapse in response to COVID-19 circumstances |
| Stream of consciousness | Shares personal experiences and/or feelings about COVID-19-related struggles |
| Triggers for use/cravings | Discusses reminders of using drugs, cravings, etc. |
| Using quarantine to get sober | Generated by coders |
| Withdrawal | Discusses experiences with withdrawal connected to COVID-19 circumstances |
| Note: This table only includes codes used to filter out relevant subreddit posts for this particular thematic analysis; it is not the comprehensive list of codes originally proposed in our larger project. | |
